# Good adherence to HAART and improved survival in a community HIV/AIDS treatment and care programme: the experience of The AIDS Support Organization (TASO), Kampala, Uganda

**DOI:** 10.1186/1472-6963-8-241

**Published:** 2008-11-20

**Authors:** Andrew M Abaasa, Jim Todd, Kenneth Ekoru, Joan N Kalyango, Jonathan Levin, Emmanuel Odeke, Charles AS Karamagi

**Affiliations:** 1 Clinical Epidemiology Unit, Makerere University, P.O. Box 7072, Kampala, Uganda; 2 Statistics Unit-Medical Research Council (MRC/UVRI), P.O. Box 49, Entebbe, Uganda; 3 Department of Pharmacy, Makerere University, P.O. Box 7072, Kampala, Uganda; 4 The Aids Support Organisation (TASO), P.O Box 10443, Kampala Uganda; 5 Department of Paediatrics and Child Health, Makerere University, P.O. Box 7072, Kampala, Uganda

## Abstract

**Background:**

Poor adherence to highly active antiretroviral therapy (HAART) may result in treatment failure and death. Most reports of the effect of adherence to HAART on mortality come from studies where special efforts are made to provide HAART under ideal conditions. However, there are few reports of the impact of non-adherence to HAART on mortality from community HIV/AIDS treatment and care programmes in developing countries. We therefore conducted a study to assess the effect of adherence to HAART on survival in The AIDS Support Organization (TASO) community HAART programme in Kampala, Uganda.

**Methods:**

The study was a retrospective cohort of 897 patients who initiated HAART at TASO clinic, Kampala, between May 2004 and December 2006. A total of 7,856 adherence assessments were performed on the data. Adherence was assessed using a combination of self-report and pill count methods. Patients who took ≤ 95% of their regimens were classified as non-adherent. The data was stratified at a CD4 count of 50 cells/mm^3^. Kaplan Meier curves and Cox proportional hazards regression models were used in the analysis.

**Results:**

A total of 701 (78.2%) patients had a mean adherence to ART of > 95%. The crude death rate was 12.2 deaths per 100 patient-years, with a rate of 42.5 deaths per 100 patient-years for non-adherent patients and 6.1 deaths per 100 patient-years for adherent patients. Non-adherence to ART was significantly associated with mortality. Patients with a CD4 count of less than 50 cells/mm^3 ^had a higher mortality (HR = 4.3; 95% CI: 2.22–5.56) compared to patients with a CD4 count equal to or greater than 50 cells/mm^3 ^(HR = 2.4; 95% CI: 1.79–2.38).

**Conclusion:**

Our study showed that good adherence and improved survival are feasible in community HIV/AIDS programmes such as that of TASO, Uganda. However, there is need to support community HAART programmes to overcome the challenges of funding to provide sustainable supplies particularly of antiretroviral drugs; provision of high quality clinical and laboratory support; and achieving a balance between expansion and quality of services. Measures for the early identification and treatment of HIV infected people including home-based VCT and HAART should be strengthened.

## Background

The introduction of HAART has greatly improved the survival of HIV/AIDS infected people. HAART reduces morbidity and mortality by suppression of viral replication, restoration and preservation of immune function, and prevention of drug resistance [1-4]. Mortality among patients on HAART is associated with high baseline levels of HIV RNA [5]; WHO stage III or IV at the beginning of treatment, low body mass index; severe anaemia; low CD4 cell count [1,6]; type of ART treatment; cotrimoxazole prophylaxis[7]; gender [8,9]; resource-poor settings [10] and poor adherence to HAART [11-13].

Adherence to HAART is critical to the survival of HIV/AIDS infected people. A pooled analysis of North American studies reported adherence of 55% (95% CI 49%–62%) while for African studies adherence was 77% (95% CI 68%–85%)[14]. Non adherence to HAART is a major public health concern because it leads to virologic, immunologic and clinical failure, and increases the risk of transmission of drug resistant virus [15]. The major causes of non adherence to HAART are forgetfulness, lack of understanding of treatment benefits, severity of adverse events, and the level of complexity of the drug regimen[14].

Given this complexity, the effect of adherence to HAART on survival has been reported mainly in clinical trials or other controlled environments [1,3,12,16]. However, due to the high demand for HAART, other avenues including community HIV/AIDS treatment and care programmes are increasingly being used to provide HAART. In Uganda, The AIDS Support Organization (TASO) was established in 1987 as the first indigenous non-governmental organization (NGO) in Africa to respond to the needs of people living with HIV/AIDS. TASO's community HAART programme was rolled out in May 2004, and by the end of December 2005, the cumulative number of TASO clients enrolled on HAART countrywide was 5,788 [17]. However, it is not known whether the TASO community HAART programme confers survival benefits that are similar to those reported in controlled or government settings. We therefore conducted a study to determine the effect of non adherence to HAART on survival in the TASO community HIV/AIDS treatment and care programme, Kampala, Uganda.

## Methods

The study was carried out in The AIDS Support Organization (TASO) Mulago clinic Kampala, Uganda. HIV/AIDS patients are identified through voluntary counselling and testing, and registered at TASO if found to be HIV positive. The criteria for HAART entry are: a CD4 cell count below 200 cells/mm^3^; WHO stage 3 or 4 disease; or a history of recurrent herpes zoster [18]. Before enrolment, the patient is asked to identify a medicine companion who is normally a relative, friend, neighbour or spouse to help with adherence, and to remind him/her to take the medicines. After enrolment, the patient is given medicines for 3 days and assessed for adherence. If he/she takes all the pills over the three days, he/she is given medicines for 2 weeks. If the adherence is still good at the end of the 2 weeks, he/she is given drugs for 2 months and thereafter for 6 months. Patients who are found to have poor adherence are given appointments for adherence counselling.

There are routine clinic visits after enrolment when HAART drugs are dispensed and adherence assessed: at two weeks; two months; and thereafter at six months. The distribution of follow-ups depends on the patient's performance in terms of improvement and adherence exhibited. The follow-ups are carried out by trained and experienced counsellors and field staff. TASO provides medical care through its clinics which are held twice a week. Pill count adherence is monitored by asking patients to retain any missed doses in their pill boxes, and pill boxes are checked when medicines are re-filled by the home visitors for patients on the home care programme and at the clinic for patients on the facility based treatment and care programme. Adherence is assessed as pill count (PCA) which is the number of pills actually taken as a percentage of the number of pills delivered for the home based treatment programme or dispensed for the facility based programme. Self reported adherence measurement technique is used by asking the patients the number of times they have missed taking their pills. The adherence levels from the two measures are compared. The higher level is recorded as the patient's adherence level for that assessment and the lower level, if less or equal to 95%, forms the basis for the patient's follow up for adherence support through adherence counselling.

All HIV/AIDS patients at TASO Mulago clinic, who were aged at least 15 years, initiated antiretroviral treatment in the period from May 2004 to December 2005, and whose records were readily available, were included in this analysis. We used combined inferences from multiple imputations to impute for the missing data in the important variables of study interest. The mean adherence to HAART for each eligible record was computed and the records were divided into two categories: adherent (average adherence greater than 95%) and non-adherent (≤ 95% adherence). Death was confirmed from death notification filled by the TASO medical team. Analysis was based on all-cause mortality.

Data was cleaned and coded using STATA release 9.0 (StataCorp, USA). Background characteristics between adherent and non-adherent patients were compared by logistic regression. The outcome of interest was survival time as measured from the date of HAART initiation to the reported date of death for patients who died, or the date of the last recorded visit for patients who were censored. Kaplan Meier plots, and log rank tests were used to illustrate survival in different groups. Cox proportional hazards regression models were used to investigate factors associated with survival. Interactions between factors were also investigated. Age, sex and predictor variables found to be significant (p < 0.05) in univariable analyses were considered as candidate regressors in the Cox models. The proportional hazards assumption was tested using Schoenfeld residuals.

### Regulatory approvals

The study was approved by Makerere University Clinical Epidemiology Unit (CEU), Makerere University Faculty of Medicine Research and Ethics Committee, The AIDS Support Organisation (TASO) Research and Ethics Committee, and the Uganda National Council for Science and Technology.

### Role of the funding sources

The funding sources had no role in the design, conduct, or reporting of the study findings or the decision to submit the manuscript for publication.

## Results

A total of 897 patients were eligible for the study and 7,856 adherence assessments were reviewed. Of these assessments, 6,527 (83%) recorded adherence > 95%. Out of the 897 patients studied, 196 (21.9%) patients had a mean adherence of 95% or less, and 701 (78.2%) patients had overall mean adherence of greater than 95% (Table 1). The median number of adherence assessments for non-adherent and adherent patients were 6.9 and 10.3 respectively.

**Table 1:** Adherence to HAART among 897 patients in TASO Kampala, Uganda

**Variable**	**Category**	**Total**	**Adherent to HAART, n (%)**	**95% CI**
**Overall**		**897**	**701 (78.2)**	**75.3–80.8**
				
**Sex**	Male	222	166 (74.8)	68.5–80.3
	Female	675	535 (79.3)	76.0–82.3
				
**Age in years**	Less than 35	301	246 (81.7)	76.9–85.9
	35 or more	596	455 (76.3)	72.7–79.7
				
**Education**	None	157	111 (70.7)	62.9–77.7
	Educated	740	590 (79.7)	76.6–82.6
				
**Religion**	Catholic	274	212 (77.4)	72.0–82.2
	Protestant	363	293 (80.7)	76.3–84.6
	Pentecostal	143	106 (74.1)	66.1–81.1
	Orthodox	54	42 (77.8)	64.4–88.0
	Muslim	56	41 (73.2)	59.7–84.2
	Other	7	7 (100)	100+
				
**Married**	Ever	866	676 (78.1)	75.2–80.8
	Never	31	25 (80.6)	62.5–92.5
				
**CD4 count**	Less than 50	197	172 (87.3)	81.8–91.6
	50 or more	700	529 (75.6)	72.2–78.7

+ One sided 97.5% CI

The majority of the patients were females (75.3%), reflecting TASO's patient distribution ratio of males to females of approximately 1:3 (Table 2). The mean age at initiation of HAART was 38.6 years (SD = 8.4) while the median age was 37 years. The majority of the patients (78%) studied were initiated on HAART with a baseline CD4 count of 50 cells/mm^3 ^or more. Most patients (96.5%) had been married while 82.5% had attained at least primary education.

**Table 2:** Background and clinical characteristics of 897 patients on HAART in TASO Kampala, Uganda

**Variable**	**Non Adherent n (%)**	**Adherent to HAART, n (%)**	**Odds Ratio**	**95% CI**
**Sex**				
Male	56 (28.6)	166 (23.7)	1.0	
Female	140 (71.4)	535 (76.3)	0.77	0.54–1.11
				
**Age in median years**	36	37	N/A	N/A
				
**Education**				
None	46 (23.5)	111 (15.8)	1.0	
Educated	150 (76.5)	590 (84.2)	0.61	0.42–0.90
				
**Religion**				
Catholic	62 (31.6)	212 (30.2)	1.0	
Protestant	70 (35.7)	293 (41.8)	1.25	0.65–2.41
Pentecostal	37 (18.9)	106 (15.1)	1.19	0.75–1.91
Orthodox	12 (6.1)	42 (6.0)	0.98	0.48–1.97
Muslim	15 (7.7)	41 (5.9)	0.82	0.56–1.20
Other	0 (0.0)	7 (1.0)	-	-
				
**Married**				
Ever	190 (96.9)	676 (96.4)	1.0	
Never	6 (3.1)	25 (3.6)	1.17	0.47–2.90
				
**CD4 count**				
Less than 50	25 (12.8)	172 (24.5)	1.0	
50 or more	171 (87.2)	529 (75.5)	2.22	1.41–3.50

The total patient-time contributed was 1,343.4 patient-years. During the study period, 164 patients (18.3%) died giving a crude death rate of 12.2 per 100 patient-years (95% CI; 10.5–14.2) (Table 3). The maximum observed exit time was approximately 2.6 years, and the last death was observed at 2.3 years. For the non adherent patients, 94 deaths were reported, giving an overall mortality rate of 43 deaths per 100 patient-years (95% CI: 35 – 52), while for adherent patients 68 deaths were reported, giving a mortality rate of 6.1 deaths per 100 patient-years (95% CI: 5 – 8). The patients lost to follow up were 147 (16.4%).

**Table 3:** Unadjusted analysis of mortality according to adherence and other characteristics among 897 patients in TASO Kampala, Uganda

**Variable**	**Total Deaths**	**Total Patient-years (Death rate/100 Patient-years)**	**HR**	**95% CI**
**Overall**	**164**	**1343.4 (12.2)**	-	**10.5–14.2**
				
**Sex**				
Male	34	333.6 (10.2)	1.0	
Female	130	1009.8 (12.9)	1.26	0.86–1.83
				
**Age in years**				
Less than 35	56	462.4 (12.1)	1.0	
35 or more	108	881.0 (12.3)	1.04	0.75–1.44
				
**Education**				
None	45	224.1 (20.1)	1.0	
Educated	119	1119.3 (10.6)	0.52	0.37–0.73
				
**Religion**				
Catholic	55	408.6 (13.5)	1.0	
Protestant	63	543.2 (11.6)	0.88	0.61–1.26
Pentecostal	29	216.5 (13.4)	1.01	0.65–1.59
Orthodox	9	82.3 (10.9)	0.82	0.40–1.65
Muslim	8	82.6 (9.7)	0.78	0.37–1.63
Other	0	10.6 (0.0)	-	-
				
**Married**				
Never	5	46.7 (10.7)	1.0	
Ever	159	1296.7 (12.3)	1.20	0.49–2.93
				
**CD4 count**				
Less than 50	86	306.7 (28.0)	1.0	
50 or more	78	1036.6 (7.5)	0.29	0.21–0.39
				
**Adherence**				
Non adherence	94	226.0 (42.5)	1.0	
Adherence	68	1117.3 (6.1)	0.11	0.08–0.16

**Figure 1:**
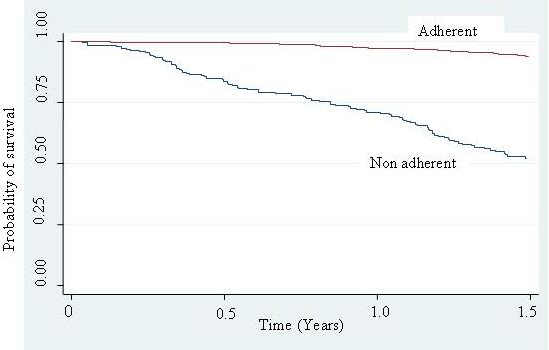
Kaplan Meier survival estimates in 897 HIV/AIDS patients initiated on HAART in TASO Kampala, Uganda, by Adherence status.

**Figure 2:**
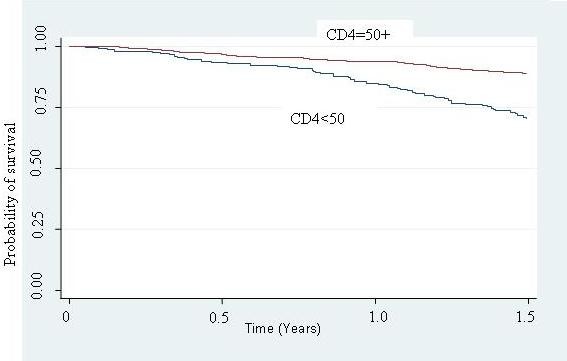
Kaplan Meier survival estimates in 897 HIV/AIDS patients initiated on HAART in TASO Kampala, Uganda, by CD4 count at initiation.

**Figure 3:**
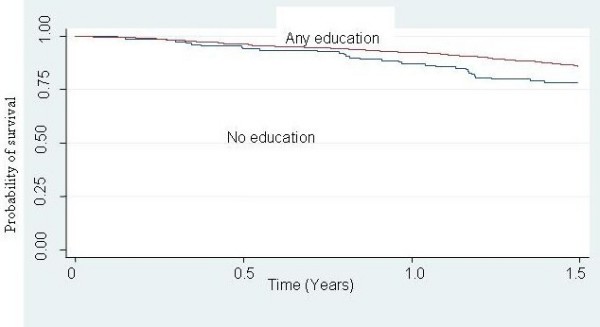
Kaplan Meier survival estimates in 897 HIV/AIDS patients initiated on HAART in TASO Kampala, Uganda, by Education status.

Table 3 shows the unadjusted survival analyses for socio-demographic and clinical characteristics. Better survival (lower hazard) was seen for those with higher education level (HR = 0.52, 95% CI 0.37–0.73), patients with baseline CD4 count of above 50 cells/mm^3 ^(HR = 0.29, 95% CI 0.21–0.39), and those with >95% adherence (HR = 0.11, 95% CI 0.08–0.16). Kaplan Meier survival curves are shown for the significant variables (Figure 1, 2, 3).

There was significant interaction between CD4 count at initiation of HAART and adherence in their effect on survival (p < 0.001) (Table 4). In patients with CD4 count less than 50 cells at HAART initiation, adherence showed a 4-fold reduction in survival (HR 0.23, 95% CI 0.18–0.45). In patients with CD4 count greater than or equal to 50 cells at HAART initiation, adherence showed a 2-fold reduction in survival (HR 0.42, 95% CI 0.31–0.56). In multivariate analysis, adherence to HAART was associated with survival (HR 0.46, 95% CI 0.47–0.50) after controlling for age, sex and education level (Table 5).

**Table 4:** Interaction between CD4 count at HAART initiation and adherence among 897 HIV/AIDS patients in TASO Kampala, Uganda

**Variable**	**CD4 count (cells)**	**Hazard Ratio**	**95% CI**
**Adherence >95%**	<50	0.23	0.18–0.45
**Adherence >95%**	≥ 50	0.42	0.31–0.56

Likelihood ratio test was significant (p < 0.001)

**Table 5:** Adjusted association between adherence and mortality among 897 patients in TASO Kampala, Uganda

**Variable**	**Category**	**Hazard Ratio**	**95% CI**
**Sex**	Female	1.48	0.98–2.17
**Age in years**	35 or more	0.93	0.67–1.29
**Education level**	Any	0.67	0.47–0.94
**Adherence**	>95%	0.46	0.47–0.50

## Discussion and Conclusion

Our study showed that the majority of the patients attending TASO Mulago clinic, Kampala, had good adherence to HAART with 78.2% of the patients achieving a mean adherence of greater than 95%. Comparison of adherence to HAART between studies is fraught with many obstacles including differences in study population and design, definition and measurement of adherence, as well as differences in sample size and type of HAART. Nevertheless, the adherence rate in our study was similar to that reported in previous studies in sub-Saharan Africa [14]. The high adherence rate to HAART observed in TASO is remarkable considering that its HAART programme is in a non research or government setting. The high adherence to HAART can be attributed to the continuity of care and close support of the patients that is the hallmark of TASO and which has also been described elsewhere [19]. TASO originated as a patient-centred care and support organization and this philosophy has persisted even after the introduction of HAART.

The overall mortality was 12 per 100 person-years but the estimate may have been affected by four major factors. Compared to settings where HAART is initiated at CD4 counts of <350 cells/mm^3 ^the patients in our study were initiated late on HAART (<200 cells/mm^3^), though it is the policy of Ministry of Health. This late initiation on HAART may have increased the mortality in our study. We also excluded participants who had been on HAART for less than one year in an effort to capture mortality that occurred only after HAART was deemed to be effective. By so doing however we may have inadvertently excluded early mortality and yet several studies have reported that mortality among HAART patients is greatest in the first three months of treatment [3,10,20]. Thus exclusion of early mortality may have led to an underestimate of overall mortality in our study.

In our study, there were difficulties in ascertaining the cause of death. The cause of death was determined by the TASO medical team and was mostly in the form of verbal autopsy which classifies deaths in broad categories but does not permit assignment of a specific cause of death [21]. Thus there were limitations in determining the underlying cause of death and we therefore performed all-cause mortality analysis. The use of all-cause mortality in our study may have led to an under or overestimation of the overall mortality among HAART patients. The use of the multiple imputation method to impute missing data may also have distorted our estimates if the missing data were not random[22]. Finally, under or overestimation of mortality could have occurred because a significant proportion (16.4%) of the participants on HAART were lost to follow up. Nevertheless, the overall mortality in our study was similar to that reported in previous studies [10,20,23-28].

Non-adherent participants had a mortality of 42.5 deaths per 100 person-years and, after adjusting for age, sex and education level, were two times as likely to die as adherent participants. These findings are consistent with other studies that have reported that non-adherence is associated with increased mortality [12,27,29]. Non adherence to HAART leads to virologic, immunologic and clinical failure that is mediated mainly by two potentially re-enforcing mechanisms. Non-adherence to HAART leads to failure to suppress viral replication, thus increasing the likelihood of developing HIV mutations that could lead to the development of drug-resistant viral strains. Secondly, non-adherence to HAART fails to prevent further viral destruction of the cellular immune system with consequent reduction in the level of CD4+ cells and development of opportunistic infections [15]. It is worth noting that mortality among adherent participants in our study (6.1 per 100 person-years) was comparable to mortality rates reported in developed world settings.

In our study we found overwhelming evidence of a statistical interaction between baseline CD4 count and non-adherence i.e. baseline CD4 count modified the effect of non-adherence. In patients with a CD4 count <50 cells/mm^3 ^non-adherence increased the risk of mortality four fold, whereas in patients with a CD4 count of 50 cells/mm^3 ^or above at baseline, non-adherence doubled the risk of mortality. Previous studies have reported that CD4 counts at the initiation of HAART independently modify survival with decrease in CD4 count being associated with increased mortality [3,6,12,27]. Although Nachega et al reported that there was no interaction between adherence and baseline CD4 count, they found that CD4 count strongly modified the effect of adherence on survival, with the greatest mortality (HR 4.54, 95% CI: 2.83–7.29) occurring in the most immunosuppressed patients (≤ 50 cell/μL.

The findings of this analysis have important policy implications particularly for HAART programmes in developing countries. Our study has shown that good adherence to HAART and improved HIV survival are feasible in a community HIV/AIDS treatment and care programme. Uganda, like many developing countries, has limited capacity to provide HAART through the government health care system. Accordingly, there is increasing need to provide HAART through non governmental programmes including home or community care programmes. As of June 2007, of the more than 100,000 people in Uganda taking HAART, 14% received their drugs through home or community care programmes, mostly through TASO.

The success of the TASO community HAART programme can be attributed to a number of factors including the personalized patient-centred approach; the continuity of care and follow up through home visits; total patient and family care and support; and free access to services. These factors have contributed to the high adherence rates in TASO that are critical for improved survival of HIV infected people. The major challenges of the TASO community HAART programme include the provision of high quality clinical and laboratory services (e.g. CD4 cell counts) to support HAART; funding to provide sustainable provision of antiretroviral drugs and other supplies; and the need to balance the expansion of the TASO community HAART programme to meet increasing demand without losing the personal touch that has over the years ensured the quality of services and success of TASO.

Our study highlighted the poorer outcomes in HIV infected people with low CD4 counts and echoes the need to identify and treat eligible HIV infected people early and for intensive adherence interventions to target HIV-1 infected patients with the lowest CD4 counts [16]. The home-based VCT programme that is also offered in TASO is one way in which HIV infected people can be identified early so that they benefit maximally from HAART.

The principal limitation of our study was the study design. The study was a retrospective cohort, and therefore we did not control for all the potential confounders that could confound the association between non-adherence to antiretroviral therapy and patients' survival, such as viral load and opportunistic infections at initiation of HAART. Viral loads were not recorded by TASO, while there was no precise information on opportunistic infections at the time of initiation of antiretroviral therapy. This situation may have resulted in an under or over estimate of the association between non adherence and survival. However, random error was unlikely to have biased our findings because the study needed to observe 121 deaths to show a significant effect in survival but we observed 164 deaths.

The measurement and definition of adherence to antiretroviral therapy may have introduced bias. There is no gold standard for measuring adherence to antiretroviral therapy [14]. The approaches employed in this study included patient self-report and pill counts. The self-report obtains a patient's subjective evaluation of his or her own level of treatment compliance behaviour. The advantages of the self-report method include its simplicity, speed, and viability of use. The disadvantages include reliance on recall and social desirability bias, with a tendency to overestimate adherence. Nevertheless, several studies highlight the usefulness of the self-report as an adherence measurement tool and show it to correlate well to virologic outcome [30,31]. Adherence measurement depended on a clinician's subjective judgment. Also using mean adherence over the period a patient was on treatment could have introduced measurement bias into our study.

In our study, the adherence assessments for non-adherent and adherent patients were unequal. One interpretation of this finding is that unequal assessment of adherence between the adherent and the non adherent patients may have introduced bias in our estimates. However, an alternative and more plausible explanation is that differences in adherence levels between patients especially in the clinic-based programme may instead have lead to differences in adherence assessments between the adherent and non adherent patients since the adherence assessments were based on patient visits to the TASO clinic. The type of regimen patients were taking could have had an impact on their survival and hence distorted our estimates. As already mentioned, we could not control for the type of regimen patients were taking. While our findings may have been affected by such factors, we believe that our overall conclusions are robust.

In conclusion, our study showed that good adherence to HAART and improved survival are feasible in community HIV/AIDS treatment and care programmes such as that of TASO, Uganda. However, there is need to support community HIV/AIDS HAART programmes to overcome the challenges of funding to provide sustainable supplies particularly of antiretroviral drugs; provision of high quality clinical and laboratory support; and achieving a balance between expansion and maintenance of quality of services. Measures for early identification and treatment of HIV infected people including home-based VCT and HAART should be strengthened.

## Competing interests

The authors declare that they have no competing interests.

## Authors' contributions

AMA participated in the conception, design, and implementation of the study, statistical analysis, interpretation and drafting of manuscript. JNK and JT participated in study design, statistical analysis, interpretation and drafting of manuscript. JL participated in the statistical analysis and interpretation of the study. KE participated in the design, statistical analysis and interpretation of the study. EO participated in the conception, design, and interpretation of the study. CASK participated in study conception, design, interpretation and drafting of manuscript. All authors read and approved the final manuscript.

## Pre-publication history

The pre-publication history for this paper can be accessed here:


